# Association of Radioactive Iodine, Antithyroid Drug, and Surgical Treatments With Solid Cancer Mortality in Patients With Hyperthyroidism

**DOI:** 10.1001/jamanetworkopen.2020.9660

**Published:** 2020-07-23

**Authors:** Cari M. Kitahara, Dale L. Preston, Julie Ann Sosa, Amy Berrington de Gonzalez

**Affiliations:** 1 Radiation Epidemiology Branch, Division of Cancer Epidemiology and Genetics, National Cancer Institute, National Institutes of Health, Bethesda, Maryland; 2 Hirosoft International, Eureka, California; 3 Department of Surgery, University of California, San Francisco

## Abstract

**Importance:**

The long-term health effects of radioactive iodine (RAI) and antithyroid drug (ATD) treatments compared with surgery for hyperthyroidism remain uncertain.

**Objective:**

To compare solid cancer mortality rates associated with RAI and ATD treatments vs surgical management for hyperthyroidism.

**Design, Setting, and Participants:**

This multicenter cohort study assessed patients treated for hyperthyroidism from January 1, 1946, to December 31, 1964, with follow-up through December 31, 2014. Data analysis was performed from August 1, 2019, to April 23, 2020.

**Exposures:**

Management with RAI, ATDs, surgical intervention, or combinations of these treatments.

**Main Outcomes and Measures:**

Comparisons of solid cancer mortality rates in each treatment group with expected rates from the general population were assessed using standardized mortality ratios (SMRs), and internal comparisons were assessed using hazard ratios (HRs) adjusted for age, sex, and underlying diagnosis (Graves disease or toxic nodular goiter).

**Results:**

Of 31 363 patients (24 894 [79.4%] female; mean [SD] age, 46.9 [14.8] years) included in the study, 28 523 (90.9%) had Graves disease. The median follow-up time was 26.0 years (interquartile range, 12.3-41.9 years). Important differences in patient characteristics existed across treatment groups at study entry. Notably, the drug-only group (3.6% of the cohort) included a higher proportion of patients with prior cancers (7.3% vs 1.9%-4.0%), contributing to an elevated SMR for solid cancer mortality. After excluding prior cancers, solid cancer SMRs were not elevated in any of the treatment groups (SMR for surgery only, 0.82 [95% CI, 0.66-1.00]; SMR for drugs only, 0.90 [95% CI, 0.74-1.09]; SMR for drugs and surgery, 0.88 [95% CI, 0.84-0.94]; SMR for RAI only, 0.90 [95% CI, 0.84-0.96]; SMR for surgery and RAI, 0.66 [95% CI, 0.52-0.85]; SMR for drugs and RAI, 0.94 [95% CI, 0.89-1.00]; and SMR for drugs, surgery, and RAI, 0.85 [95% CI, 0.75-0.96]), and no significant HRs for solid cancer death were observed across treatment groups. Among RAI-treated patients, HRs for solid cancer mortality increased significantly across levels of total administered activity (1.08 per 370 MBq; 95% CI, 1.03-1.13 per 370 MBq); this association was stronger among patients treated with only RAI (HR, 1.19 per 370 MBq; 95% CI, 1.09-1.30 per 370 MBq).

**Conclusions and Relevance:**

After controlling for known sources of confounding, the study found no significant differences in the risk of solid cancer mortality by treatment group. However, among RAI-treated patients, a modest positive association was observed between total administered activity and solid cancer mortality, providing further evidence in support of a dose-dependent association between RAI and solid cancer mortality.

## Key Points

**Question:**

Are radioactive iodine or antithyroid drug treatments for hyperthyroidism associated with greater risk of solid cancer mortality compared with surgical management?

**Findings:**

In this cohort study of 31 363 patients who were cancer free at baseline, no association was found between treatment group (radioactive iodine, medications, and/or surgery) and risk of solid cancer mortality. Among patients receiving radioactive iodine treatment, the association with solid cancer mortality increased with greater total administered activity.

**Meaning:**

The findings suggest that the association between radioactive iodine treatment and solid cancer mortality is dose dependent.

## Teaser

This cohort study compares the association of radioactive iodine or antithyroid drug treatments for hyperthyroidism vs surgical management with solid cancer mortality in patients without cancer.

## Introduction

Overt hyperthyroidism is diagnosed in approximately 0.5% of the US population.^1^ The most common diagnosis is Graves disease, with a lifetime prevalence of approximately 3% for women and less than 1% for men. Radioactive iodine (RAI), antithyroid drugs (ATDs), and thyroidectomy alone or in combination are the 3 main modalities used in the management of hyperthyroidism; however, treatment practices vary substantially over time and by geographic region. For toxic nodular goiter (TNG), RAI and thyroidectomy are the primary treatment options, with long-term ATD treatment offered less frequently.^1^ For uncomplicated Graves disease, RAI has been the treatment of choice in the US, whereas ATDs have been the more commonly preferred option in most other regions of the world.^2,3,4^ However, in the US and Europe, RAI treatment of Graves disease has been decreasing in recent decades in favor of ATDs.^2^ Evidence of long-term risks of leukemia and other secondary malignant tumors after postsurgical adjuvant treatment of differentiated thyroid cancer with RAI may have contributed to this trend.^5,6,7,8,9^

Studies evaluating the long-term adverse effects of RAI and other treatment options for hyperthyroidism may help to better inform clinical treatment practices and guidelines. Because of the lack of large randomized clinical trials, information on long-term associations of treatment with cancer and other outcomes has, thus far, relied on results from observational studies. However, results from the few large observational studies^10,11,12,13,14,15,16^ on this topic have been inconsistent, and summarizing and comparing the findings across studies are challenging because of differences in sample size, duration of follow-up, information on treatment types and doses, statistical methods, and ability to control for confounding and other sources of bias. Confounding is especially of concern for studies that rely on an external comparison group (eg, general population rates) because patients can differ in many important ways from the general public in terms of genetic, environmental, and behavioral factors and access to care. In addition, the underlying disease itself may be directly associated with risk of the outcome under study independently of any treatment effects.^17,18^ Another source of bias is *confounding by indication* (or *treatment allocation bias*) in which the reason or choice of one treatment vs another is associated with the baseline risk of the outcome under study (eg, patients’ age, sex, general health status, and disease origin and severity).^19,20^ Thus, although some of these studies have found increased incidence or mortality from cancers of the stomach,^11,12,13^ kidney,^12^ breast,^12^ and thyroid^10,14^ among RAI-treated patients, the direct effects of treatment are difficult to discern in the presence of uncontrolled confounding.

With use of data from the Cooperative Thyrotoxicosis Therapy Follow-up Study (CTTFUS), a large cohort study of patients treated for hyperthyroidism between the 1940s and 1960s in the US and UK, a positive association between organ-absorbed dose and mortality from solid cancers (including breast cancer) was previously reported.^21^ The focus on RAI-treated patients was a strength because it helped to minimize confounding caused by imbalances in risk factors across treatment groups and provided a direct assessment of the risk per unit dose.^22^ Nonetheless, comparisons of risks across treatment groups are of interest for practitioners involved in the management of hyperthyroidism who must select the treatment modality and the extent of treatment.

Thus, for the current study, we used data from the CTTFUS cohort to evaluate the association between treatment received (RAI, ATDs, surgery, and various combinations of these) and risk of solid cancer death. We also investigated these risks across levels of RAI-administered activity (as a more intuitive measure and proxy for organ-absorbed doses). Results presented in the current article informed the methodologic approach used in a recent analysis^21^ of organ-absorbed dose. With an additional 24 years of follow-up, the current study also provides an update of the standardized mortality ratios (SMRs) for solid cancer published by Ron et al^10^ more than 2 decades ago.

## Methods

### Study Population

This cohort study included data from the original CTTFUS cohort, which included 35 593 patients with hyperthyroidism treated with RAI, ATDS, surgery, or some combination of these at 25 US and 1 British medical centers between January 1, 1946, and December 31, 1964.^10^ Comprehensive clinical data were abstracted from medical records, and early follow-up information was obtained from treating physicians, clinics, or patients when no medical sources were available. In 1984, the National Cancer Institute and 4 regional study centers reassembled patient and treatment data from computer lists, microfiche, microfilm cassettes, and handwritten material; all but 4010 patients (n = 31 583) were eligible for subsequent tracing and mortality follow-up through 1990.^10^ Follow-up of US patients was recently extended through December 31, 2014.^21^ Person-time for all patients was defined as the date of the first study examination through the date of death, date of loss to follow-up or last known vital status, or end of study, whichever occurred first. This study was approved by the institutional review board of the National Cancer Institute of the National Institutes of Health. Because mortality follow-up was based on linkages with available databases and involved no direct contact with study participants, the requirement for informed consent was waived. All data were deidentified. This study followed the Strengthening the Reporting of Observational Studies in Epidemiology (STROBE) reporting guideline.

For the current analyses, we additionally excluded patients with no follow-up time (n = 206), those with missing entry or exit dates (n = 8), and those whose exit dates occurred on or before the study entry date (n = 6) (Figure). Of the 31 363 remaining patients, death records were available for 25 445 (81.1%), whereas 2829 (9.0%) were censored at their last known vital status date and the others were found or presumed to be alive by the end of follow-up on December 31, 2014 (3089 [9.8%]). Data analysis was performed from August 1, 2019, to April 23, 2020.

**Figure.**
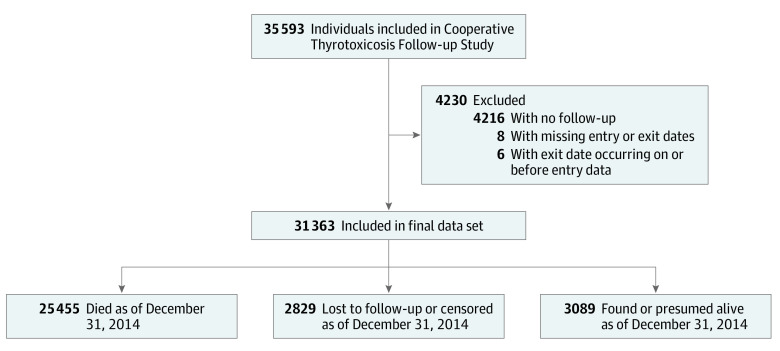
Description of the Cooperative Thyrotoxicosis Therapy Follow-up Study and Inclusion and Exclusion Criteria for the Current Analysis

### Statistical Analysis

For external comparisons, SMRs and 95% CIs were used to compare the observed number of solid cancer (including breast and nonbreast solid cancer) deaths in the cohort with the expected number of deaths based on age-, sex-, and calendar year–specific mortality rates in the general populations of the US and UK. The SMRs were calculated separately by treatment group. Influence of possible sources of bias were examined through exclusion of specific patient groups (eg, those with comorbidities) or person-time (to account for potential exposure-disease latency periods).

For internal cohort comparisons, Cox proportional hazards regression models were used to estimate multivariable-adjusted hazard ratios (HRs) and 95% CIs for the association between treatment groups (or categories of total administered activity) and risk of solid cancer death. All models excluded patients with prior cancer at study entry (n = 1013) or between entry and date of last RAI treatment (n = 36), used attained age as the time scale (incorporating a 5-year latency period), and were adjusted for sex and underlying diagnosis (Graves disease, TNG, and other or unknown). Statistical analyses were conducted with Stata, version 16.0 (StataCorp) and Epicure, version 2.00.02 (Risk Sciences International). A 2-sided *P* < .05 was considered to be statistically significant.

## Results

### Cohort Characteristics

A total of 31 363 patients (24 894 [79.4%] female; mean [SD] age, 46.9 [14.8] years) were included in the study. Baseline characteristics of CTTFUS patients (1946-1964) are described in Table 1. A total of 28 523 patients (90.9%) received a diagnosis of Graves disease. Use of ATDs (alone or in combination with other treatments) was recorded for 22 357 patients (71.3%), RAI for 19 589 patients (62.5%), and surgery for 13 676 patients (43.6%). Whereas 7474 (23.8%) were treated with only RAI, 1138 (3.6%) were treated using only drugs and 800 (2.6%) underwent surgery. Data on ATD types, dosages, and treatment dates were unavailable or largely incomplete. For 2718 patients (89.0%) treated with both surgery and RAI, the date of surgery preceded the date of first RAI treatment, suggesting that these were mostly patients undergoing subtotal thyroidectomy who subsequently experienced relapse. Among RAI-treated patients, 12 979 (66.3%) received 1 round of treatment, whereas 6610 (33.7%) received multiple rounds. Median administered activity was 270.8 MBq (interquartile range [IQR], 187.8-422.5 MBq) for Graves disease, 488.1 MBq (IQR, 300.1-817.7 MBq) for TNG, 373.4 MBq (IQR, 233.3-569.9 MBq) for other or unknown, and 296.2 MBq (IQR, 188.7-446.2 MBq) for RAI-treated patients overall. Treatment groups differed by age, underlying diagnosis, and baseline prevalence of comorbidities. A total of 4990 patients (50.8%) in the drugs and surgery group were younger than 40 years compared with 10 487 (33.4%) overall. The surgery-only group included a high proportion of patients with TNG (356 [44.5%] vs 2491 [7.9%] overall), which accounted for the higher mean (SD) age at entry (50.8 [13.9] vs 46.9 [14.8] years). The drug-only group had the highest proportion with comorbidities, including cancer (83 [7.3%] vs 1015 [3.2%] overall).

**Table 1.** Baseline Characteristics of the Cooperative Thyrotoxicosis Therapy Follow-up Study Participants by Treatment Combination^a^

Characteristic	Total	Surgery only	Drugs only	Drugs and surgery	RAI only	Surgery and RAI	Drugs and RAI	Drugs, surgery, and RAI
Total	31 363 (100)	800 (2.6)	1138 (3.6)	9817 (31.3)	7474 (23.8)	713 (2.3)	9056 (28.9)	2346 (7.5)
Sex								
Male	6469 (20.6)	161 (20.1)	208 (18.3)	1824 (18.6)	1658 (22.2)	120 (16.8)	2107 (23.3)	386 (16.5)
Female	24 894 (79.4)	639 (79.9)	930 (81.7)	7993 (81.4)	5816 (77.8)	593 (83.2)	6949 (76.7)	1960 (83.6)
Underlying diagnosis								
Graves disease	28 523 (90.9)	436 (54.5)	1047 (92.0)	8704 (88.7)	6908 (92.5)	656 (92.0)	8500 (93.9)	2254 (96.1)
Toxic nodular goiter	2491 (7.9)	356 (44.5)	64 (5.6)	1071 (10.9)	452 (6.1)	47 (6.6)	433 (4.8)	67 (2.9)
Other or unknown	349 (1.1)	8 (1.0)	27 (2.3)	42 (0.4)	114 (1.5)	10 (1.4)	123 (1.3)	25 (1.1)
Age at study entry, y								
<40	10 487 (33.4)	182 (22.8)	468 (41.1)	4990 (50.8)	1645 (22.0)	183 (25.7)	2131 (23.5)	880 (37.5)
40-59	14 343 (45.7)	380 (47.5)	392 (34.5)	3673 (37.4)	3769 (50.4)	388 (54.4)	4573 (50.5)	1158 (49.4)
≥60	6533 (20.8)	238 (29.8)	278 (24.4)	1154 (11.8)	2060 (27.6)	142 (19.9)	2352 (26.0)	308 (13.1)
Age, mean (SD), y	46.9 (14.8)	50.8 (13.9)	45.6 (17.3)	41.0 (14.6)	50.8 (13.9)	48.8 (12.8)	50.2 (13.9)	44.8 (13.3)
Comorbidities								
Cancer	1015 (3.2)	28 (3.5)	83 (7.3)	185 (1.9)	273 (3.7)	18 (2.5)	359 (4.0)	69 (2.9)
Coronary heart disease	1794 (5.7)	32 (4.0)	109 (9.6)	205 (2.1)	569 (7.6)	38 (5.3)	743 (8.2)	97 (4.1)
Infectious disease	2108 (6.7)	25 (3.1)	124 (10.9)	467 (4.8)	501 (6.7)	33 (4.6)	814 (9.0)	144 (6.1)
Diabetes	1671 (5.3)	54 (6.8)	111 (9.8)	470 (4.8)	371 (5.0)	36 (5.1)	545 (6.0)	84 (3.6)
Respiratory disease	1422 (4.5)	15 (1.9)	84 (7.4)	288 (2.9)	357 (4.8)	17 (2.4)	556 (6.1)	105 (4.5)
Digestive system disease	2354 (7.5)	47 (5.9)	102 (9.0)	436 (4.4)	692 (9.3)	58 (8.1)	849 (9.4)	167 (7.1)
Genital urinary disease	1543 (4.9)	28 (3.5)	71 (6.2)	320 (3.3)	401 (5.4)	33 (4.6)	579 (6.4)	111 (4.7)
Nervous system disease	727 (2.3)	9 (1.1)	48 (4.2)	115 (1.2)	233 (3.1)	19 (2.7)	254 (2.8)	48 (2.1)

Abbreviation: RAI, radioactive iodine.

^a^ Data are presented as number (percentage) of patients unless otherwise indicated.

Median follow-up time was 26.0 years (IQR, 12.3-41.9 years). Duration of follow-up and proportion of early censoring differed across treatment groups (Table 2), with the shortest follow-up occurring in the drug-only group (median, 20.2 years; IQR, 5.4-41.3 years) and the longest in the drugs and surgery group (median, 33.6 years; IQR, 18.4-50.5 years). The drug-only group had the highest proportion of early censoring (135 [11.9%] vs 27 [3.4%] to 916 [10.1%]). The drug-only and drugs and surgery groups also had the highest proportion of cancer-related deaths (160 [18.3%] to 1413 [18.6%] vs 78 [13.0%] to 1253 [16.7%]). However, 59 cancer deaths (36.9%) in the drug-only group occurred in the first 5 years of follow-up compared with 74 (5.2%) in the drugs and surgery group and 10 (12.8%) to 193 (15.4%) in the other treatment groups. Overall, 59 (5.2%) in the drug-only group died of cancer in the first 5 years of follow-up vs 74 (0.8%) to 193 (2.1%) in the other groups.

**Table 2.** Mortality Follow-up of the Cooperative Thyrotoxicosis Therapy Follow-up Study Participants by Treatment Combination^a^

Variable	Surgery only	Drugs only	Drugs and surgery	RAI only	Surgery and RAI	Drugs and RAI	Drugs, surgery, and RAI
Total	800 (2.6)	1138 (3.6)	9817 (31.3)	7474 (23.8)	713 (2.3)	9056 (28.9)	2346 (7.5)
Follow-up, median (IQR), y^b^	23.7 (13.0-39.9)	20.2 (5.4-41.3)	33.6 (18.4-50.5)	22.3 (9.7-36.9)	25.9 (11.8-39.0)	22.0 (9.7-36.3)	28.2 (14.9-43.5)
Vital status on December 31, 2014							
Lost to follow-up^c^	27 (3.4)	135 (11.9)	789 (8.0)	712 (9.5)	61 (8.6)	916 (10.1)	189 (8.1)
Presumed alive	62 (7.8)	130 (11.4)	1417 (14.4)	567 (7.6)	52 (7.3)	653 (7.2)	207 (8.8)
Died	711 (88.9)	873 (76.7)	7611 (77.5)	6195 (82.9)	600 (84.2)	7487 (82.7)	1950 (83.1)
Deaths from cancer	109 (15.3)	160 (18.3)	1413 (18.6)	1034 (16.7)	78 (13.0)	1253 (16.7)	313 (16.1)
Deaths from cancer in first 5 y of follow-up	15 (13.8)	59 (36.9)	74 (5.2)	152 (14.7)	10 (12.8)	193 (15.4)	42 (13.4)

Abbreviations: IQR, interquartile range; RAI, radioactive iodine.

^a^ Data are presented as number (percentage) of patients unless otherwise indicated.

^b^ Time in years from study entry date to end of follow-up.

^c^ Censored on date of last known vital status.

### External Comparisons 

The SMRs by treatment group are given in Table 3 and eTable 1 in the Supplement. The drug-only group had elevated mortality rates for solid cancer (SMR, 1.31; 95% CI, 1.11-1.53), breast cancer (SMR, 1.85; 95% CI, 1.31-2.62), and nonbreast solid cancer (SMR, 1.22; 95% CI, 1.01-1.46). Elevated breast cancer mortality was observed in the drugs and RAI group (SMR, 1.16; 95% CI, 1.00-1.35). Excluding patients with prior cancers attenuated these risks. Additional exclusions (patients with TNG in the first 5 years of follow-up) had little additional influence on these results. The SMRs were not elevated in the other treatment groups. The elevated solid cancer mortality rate in the drug-only group was restricted to the first 5 years of follow-up and was no longer apparent after excluding prior cancers (eTable 1 in the Supplement). After excluding prior cancers, no consistent patterns by treatment type were observed for the other solid cancers (eTable 2 in the Supplement) apart from elevated thyroid cancer mortality in RAI-treated patients, which was no longer apparent after excluding the first 5 years of follow-up and patients with TNG.

**Table 3.** SMRs by Treatment Combination Based on Ratio of Observed Numbers of Deaths Among Patients With Hyperthyroidism vs Expected Numbers of Deaths in the General Population^a^

Variable	All patients		Excluding							
			Patients with TNG		Patients with prior cancers		First 5 y of follow-up		First 5 y of follow-up and patients with TNG or prior cancers	
	Deaths, No.	SMR (95% CI)	Deaths, No.	SMR (95% CI)	Deaths, No.	SMR (95% CI)	Deaths, No.	SMR (95% CI)	Deaths, No.	SMR (95% CI)
**Solid cancer mortality**										
Surgery only	99	0.88 (0.72-1.07)	59	0.85 (0.66-1.10)	90	0.82 (0.66-1.00)	86	0.84 (0.68-1.04)	51	0.80 (0.60-1.05)
Drugs only	149	1.31 (1.11-1.53)	138	1.24 (1.05-1.47)	101	0.90 (0.74-1.09)	94	0.91 (0.74-1.11)	87	0.87 (0.70-1.07)
Drugs and surgery	1282	0.91 (0.87-0.97)	1150	0.90 (0.85-0.96)	1222	0.88 (0.84-0.94)	1217	0.92 (0.87-0.97)	1078	0.90 (0.84-0.95)
RAI only	946	1.02 (0.96-1.09)	894	1.01 (0.94-1.08)	818	0.90 (0.84-0.96)	804	0.96 (0.90-1.03)	728	0.92 (0.86-0.99)
Surgery and RAI	68	0.72 (0.56-0.91)	63	0.71 (0.55-0.91)	62	0.66 (0.52-0.85)	60	0.69 (0.54-0.89)	56	0.70 (0.54-0.91)
Drugs and RAI	1155	1.05 (0.99-1.12)	1104	1.04 (0.98-1.10)	1008	0.94 (0.89-1.00)	981	1.00 (0.94-1.07)	897	0.96 (0.90-1.03)
Drugs, surgery, and RAI	282	0.92 (0.81-1.03)	274	0.91 (0.81-1.02)	255	0.85 (0.75-0.96)	243	0.87 (0.77-0.99)	232	0.86 (0.76-0.98)
**Female breast cancer mortality**										
Surgery only	23	1.45 (0.96-2.18)	12	1.26 (0.71-2.22)	19	1.23 (0.78-1.92)	19	1.35 (0.86-2.11)	10	1.16 (0.62-2.15)
Drugs only	32	1.85 (1.31-2.62)	31	1.85 (1.30-2.63)	18	1.06 (0.67-1.68)	20	1.28 (0.83-1.99)	17	1.12 (0.70-1.81)
Drugs and surgery	213	1.03 (0.90-1.18)	186	0.99 (0.86-1.14)	196	0.96 (0.83-1.10)	201	1.04 (0.91-1.20)	172	0.98 (0.84-1.14)
RAI only	142	1.11 (0.94-1.30)	133	1.09 (0.92-1.29)	108	0.86 (0.71-1.04)	117	1.04 (0.86-1.24)	96	0.90 (0.74-1.10)
Surgery and RAI	15	1.07 (0.64-1.77)	15	1.14 (0.69-1.89)	14	1.01 (0.60-1.71)	14	1.12 (0.66-1.90)	14	1.22 (0.72-2.05)
Drugs and RAI	173	1.16 (1.00-1.35)	168	1.17 (1.01-1.36)	137	0.95 (0.80-1.12)	145	1.12 (0.95-1.32)	129	1.05 (0.89-1.25)
Drugs, surgery, and RAI	49	1.02 (0.77-1.35)	47	1.00 (0.75-1.33)	42	0.90 (0.66-1.20)	42	0.99 (0.73-1.34)	40	0.98 (0.72-1.34)
**Solid cancer mortality excluding breast**										
Surgery only	76	0.79 (0.63-0.99)	47	0.79 (0.59-1.05)	71	0.75 (0.59-0.94)	67	0.76 (0.60-0.97)	41	0.74 (0.54-1.00)
Drugs only	117	1.22 (1.01-1.46)	107	1.14 (0.95-1.38)	83	0.87 (0.70-1.09)	74	0.84 (0.67-1.06)	70	0.82 (0.65-1.04)
Drugs and surgery	1069	0.90 (0.85-0.95)	964	0.89 (0.84-0.95)	1026	0.87 (0.82-0.93)	1016	0.90 (0.85-0.96)	906	0.88 (0.83-0.94)
RAI only	804	1.00 (0.94-1.08)	761	0.99 (0.92-1.07)	710	0.90 (0.84-0.97)	687	0.95 (0.88-1.02)	632	0.92 (0.85-0.99)
Surgery and RAI	53	0.66 (0.50-0.86)	48	0.63 (0.48-0.84)	48	0.60 (0.45-0.80)	46	0.62 (0.47-0.83)	42	0.61 (0.45-0.83)
Drugs and RAI	982	1.03 (0.97-1.10)	936	1.02 (0.95-1.08)	872	0.94 (0.88-1.00)	836	0.98 (0.92-1.05)	768	0.95 (0.88-1.02)
Drugs, surgery, and RAI	233	0.90 (0.79-1.03)	227	0.90 (0.79-1.02)	213	0.84 (0.74-0.96)	201	0.85 (0.74-0.98)	192	0.85 (0.73-0.97)

Abbreviations: RAI, radioactive iodine; SMR, standardized mortality ratio; TNG, toxic nodular goiter.

^a^ Using age-, sex-, calendar period (5-year period)–, and country (US and UK) –specific strata.

### Internal Comparisons

After excluding prior cancers and adjusting for patient age, sex, and underlying diagnosis, we found no significant differences in the HR of solid cancer death across treatment groups (Table 4). Adjustment for physician-reported clinical severity of disease had almost no effect on these results (HR for drugs only, 0.98; 95% CI, 0.79-1.21; HR for surgery only, 0.85, 95% CI, 0.68-1.07; and HR for drugs and surgery, 0.98, 95% CI, 0.91-1.05). Among RAI-treated patients, risk of solid cancer death increased with total administered activity (HR, 1.08 per 370 MBq; 95% CI, 1.03-1.13 per 370 MBq; *P* = .001 for trend). Similar exposure-response patterns were observed for mortality from breast cancer (HR, 1.10 per 370 MBq, 95% CI, 0.99-1.22 per 370 MBq; *P* = .08 for trend) and nonbreast solid cancer (HR, 1.08 per 370 MBq; 95% CI, 1.03-1.13 per 370 MBq; *P* = .003 for trend). Results were stronger among patients receiving only RAI (no additional ATDs or surgery): HRs were 1.19 per 370 MBq (95% CI, 1.09-1.30 per 370 MBq) for solid cancer, 1.28 per 370 MBq (95% CI, 1.03-1.59 per 370 MBq) for breast cancer, and 1.18 (95% CI, 1.07-1.29 per 370 MBq) for nonbreast solid cancer mortality. None of the findings above changed notably after restricting the findings to patients with Graves disease.

**Table 4.** HRs for Cancer Deaths by Treatment Combinations and RAI Administered Activity Among Patients With Hyperthyroidism and No History of Cancer at Study Entry or Before Last RAI Treatment

Variable	Solid cancer mortality			Breast cancer mortality			Solid cancer mortality excluding breast		
	Deaths	HR (95% CI)^a^	*P* value	Deaths	HR (95% CI)^a^	*P* value	Deaths	HR (95% CI)^a^	*P* value
**Full population**									
By treatment group									
Surgery only	84	0.87 (0.69-1.09)	.22	18	1.20 (0.73-1.96)	.48	66	0.81 (0.63-1.04)	.10
Drugs only	90	0.97 (0.79-1.20)	.81	17	1.09 (0.67-1.78)	.73	73	0.95 (0.75-1.20)	.67
Drugs and surgery	1183	0.97 (0.90-1.04)	.40	190	0.96 (0.80-1.15)	.65	993	0.97 (0.90-1.05)	.47
RAI	1984	1 [Reference]	NA	291	1 [Reference]	NA	1693	1 [Reference]	NA
By RAI administered activity									
Not treated with RAI	1359	1.06 (0.94-1.18)	.35	225	1.20 (0.89-1.62)	.24	1134	1.03 (0.91-1.17)	.61
>0 to 185 MBq	381	1 [Reference]	NA	53	1 [Reference]	NA	328	1 [Reference]	NA
>185 to <370 MBq	938	1.10 (0.97-1.23)	.14	135	1.21 (0.88-1.67)	.23	803	1.08 (0.95-1.22)	.27
≥370 MBq	665	1.16 (1.02-1.32)	.02	103	1.40 (1.00-1.94)	.05	562	1.12 (0.98-1.29)	.10
Per 370 MBq	1984	1.08 (1.03-1.13)	.001	291	1.10 (0.99-1.22)	.08	1693	1.08 (1.03-1.13)	.003
Per 370 MBq in patients treated with RAI only	759	1.19 (1.09-1.30)	<.001	104	1.28 (1.03-1.59)	.03	655	1.18 (1.07-1.29)	.001
**Patients with Graves disease **									
By treatment group									
Surgery only	51	0.88 (0.67-1.16)	.36	10	1.19 (0.63-2.24)	.59	41	0.83 (0.61-1.13)	.23
Drugs only	85	0.95 (0.77-1.18)	.66	17	1.14 (0.70-1.85)	.61	68	0.91 (0.72-1.17)	.47
Drugs and surgery	1076	0.97 (0.90-1.05)	.50	172	0.97 (0.80-1.17)	.74	904	0.98 (0.90-1.06)	.56
RAI	1896	1 [Reference]	NA	275	1 [Reference]	NA	1621	1 [Reference]	NA
By RAI administered activity									
Not treated with RAI	1214	1.06 (0.95-1.19)	.31	199	1.18 (0.87-1.60)	.29	1015	1.04 (0.92-1.18)	.52
>0 to 185 MBq	374	1 [Reference]	NA	53	1 [Reference]	NA	321	1 [Reference]	NA
>185 to <370 MBq	908	1.10 (0.97-1.24)	.13	132	1.21 (0.88-1.67)	.24	776	1.08 (0.95-1.23)	.26
≥370 MBq	614	1.17 (1.03-1.33)	.02	90	1.32 (0.94-1.85)	.11	524	1.14 (0.99-1.31)	.06
Per 370 MBq	1896	1.07 (1.02-1.12)	.004	275	1.08 (0.96-1.22)	.19	1621	1.07 (1.01-1.12)	.01
Per 370 MBq in patients treated with RAI only	720	1.18 (1.08-1.30)	.001	94	1.28 (1.00-1.66)	.05	626	1.17 (1.06-1.29)	.003

Abbreviations: HR, hazard ratio; RAI, radioactive iodine.

^a^ Models adjusted for attained age (time scale), sex, and underlying diagnosis (Graves disease, toxic nodular goiter, and other or unknown). Models restricted to patients with Graves disease were adjusted for attained age (time scale) and sex. Follow-up began 5 years after study entry and/or at last RAI treatment.

## Discussion

Using data from a large cohort of patients treated for hyperthyroidism between the mid-1940s and mid-1960s, we evaluated the association between treatment received (RAI, ATDs, surgery, or combinations) and long-term risk of solid cancer death. After accounting for known sources of bias, we observed no significant differences in risk across treatment groups based on external and internal cohort comparisons, and notably, we found no evidence of an increased risk of solid cancer death associated with use of ATDs. Among RAI-treated patients, however, total administered activity was positively associated with risks of all solid cancers, female breast cancer, and nonbreast solid cancer death, with increases of 8% to 10% per 370 MBq. The direction and magnitude of these associations were consistent with previous findings based on estimated organ-absorbed doses.^21^

Caution is needed when comparing risks across treatment groups using observational study data, particularly when relying on general population rates as the comparison group. SMRs may be less than, equal to, or greater than unity (1.0) for reasons other than the treatment (eg, genetic factors, the disease, environmental and behavioral factors, and access to care) and should be interpreted in the context of other SMRs. Internal comparisons (eg, HR estimates) provide greater reassurance against bias by restricting comparisons to individuals with the same disease and allowing for covariate adjustment. Nonetheless, these approaches yielded similar results after accounting for major known sources of confounding.

Although some have argued that the lack of an unexposed comparison group was an important limitation of the previous analysis of organ and tissue absorbed doses and cancer mortality,^23,24,25^ the results of the current analysis suggest that the focus on RAI-treated patients was actually a strength because their inclusion could have biased our estimates of the radiation-associated risks.^26^ For instance, the surgery-only group included a higher proportion of TNG than Graves disease diagnoses (44.5% vs 7.9% in the full cohort), and the drug-only group included a high proportion of patients with baseline comorbidities, including prior cancer diagnoses, raising the possibility that these groups may have had different baseline risks of cancer death for reasons unrelated to the treatments. The elevated solid cancer mortality rate in the drug-only group, observed previously by Ron et al,^10^ has been suggested to indicate direct association of ATDs with cancer risk^23,24,25^; however, confounding by indication seems to be the more likely explanation. As was demonstrated in the earlier study,^10^ this association was no longer apparent after excluding patients with prior cancer diagnoses, and no association was observed after the first 5 years of follow-up. In addition, similar elevations in solid cancer mortality were not observed in the drugs and surgery, drugs and RAI, or drugs, surgery, and RAI treatment groups. Although numerous changes have occurred in the management of hyperthyroidism since the 1940s to 1960s, it seems plausible that patients presenting with multiple comorbidities or short life expectancies were more likely to have been managed using only ATDs rather than RAI and/or surgery. Although excluding prior cancers helped minimize this bias, it also negatively biased all the SMRs to some extent (as can be observed in Table 3) because the same exclusion could not be performed for the expected (general population) rates.

More generally, the CTTFUS cohort is not well suited to the evaluation of long-term risks associated with ATDs. The ATD formulations have changed over time, as methimazole has replaced propylthioracil as the preferred option for most patients with Graves disease because of evidence of adverse effects of propylthioracil on the liver.^27,28^ Propylthioracil was likely the most commonly prescribed ATD at the start of cohort follow-up. As described previously,^10^ no information was abstracted or retained from the medical records of these patients that would allow us to distinguish between the specific ATD types or to reconstruct the prescribed doses, and we had incomplete (and sometimes inconsistent) information on drug treatment starting and stopping dates. Considering the temporal changes in ATD treatment, more contemporary cohort studies are needed to evaluate the long-term adverse effects of methimazole specifically.

Ionizing radiation, however, is an established carcinogen.^29^ The breast is 1 of the most radiosensitive cancer sites, with epidemiologic studies^29,30,31,32,33,34^ finding consistent positive associations between absorbed dose to the breast, even in the low-dose range, and subsequent risk of breast cancer. In contrast to ATDs, data on RAI treatment dates and administered activity were nearly complete for this cohort. A previous study^21^ estimated that risks of solid cancer increased by 6%, breast cancer by 12%, and nonbreast solid cancer death by 5% for every 100-mGy absorbed dose to the stomach or breast. Similar estimates of 8% to 10% increased risk per 370 MBq were obtained in the current study using administered activity instead of organ doses (considering that 370 MBq corresponds to a median stomach dose of approximately 150 mGy [IQR, 140-170 mGy] and median breast dose of approximately 140 mGy [IQR, 120-160 mGy]).^21^ However, risk estimates based on organ doses should be more reliable because organ dose estimates accounted for clinical parameters, such as thyroid mass and percentage of uptake, in addition to administered activity.^35^ Previous estimates of risk per unit organ dose were consistent with those from other observational studies of radiation-exposed populations.^30,31,32,33,34^ In the current study, the lack of discernible differences in solid cancer risk across treatment groups may be partly explained by the low and narrow range of administered activities (median, 270.8 MBq [IQR, 187.8-422.5 MBq] for Graves disease and 488.1 MBq [IQR, 300.1-817.7 MBq] for TNG). Applying our estimates to currently recommended levels of RAI treatment (typically 370-555 MBq from a single application),^1^ the recent previous analysis^21^ estimated that 20 to 30 excess solid cancers may occur per 1000 patients treated as a direct result of the radiation exposure, although most excess deaths would likely occur more than 20 years after initial treatment. Thus, for many patients, the benefits of RAI may outweigh these risks.

### Limitations

This study has limitations. Those not already mentioned include lack of information on some potential confounding factors, such as cigarette smoking, obesity, and reproductive factors^21,26^; however, we found no association between treatment type (or radiation-absorbed dose to the lung^21^) and lung cancer mortality, suggesting that a major smoking-related bias did not occur. We lacked laboratory measures of thyroid function to assess severity of the underlying disease. The reliance on cancer mortality, as opposed to incidence, follow-up was another limitation because we could not distinguish between factors associated with cancer development vs survival. In addition, mortality follow-up is not ideal for capturing cancers with high survival rates (eg, thyroid or breast) or for studying cancer subtypes. Findings related to RAI treatment may not be generalizable to patients with thyroid cancer, who typically receive higher administered activities (>3700 MBq) in the context of postsurgical remnant ablation or therapy for presumed or known advanced or metastatic disease, potentially yielding higher or lower doses to individual organs or tissues, depending on the size and location of the residual thyroid tissue.^36^

## Conclusions

This study found positive associations between total administered activity of RAI and risks of death from total solid cancer, female breast cancer, and nonbreast solid cancers. These findings were consistent with earlier results from this cohort based on organ-absorbed doses.^21^ The low, narrow range of total administered activity and the potential for residual confounding by risk factors not captured in this study may have hindered our ability to detect differences in risk between RAI-treated and non–RAI-treated patients. No evidence supporting an association between ATD treatment and risk of solid cancer death was found in this cohort.

## Notes

Supplement. **eTable 1.** Standardized Mortality Ratios (SMRs) and 95% Confidence Intervals (CIs) for Solid Cancer Mortality by Treatment Combination, Based on Ratio of Observed Numbers of Deaths Among Patients With Hyperthyroidism Versus Expected Numbers of Deaths in the General Population, by Time Between Study Entry and Death **eTable 2.** Standardized Mortality Ratios (SMRs) by Treatment Combination, Based on Ratio of Observed Numbers of Deaths Among Patients With Hyperthyroidism Versus Expected Numbers of Deaths in the General Population, Excluding Patients With Prior Cancers
