# Supplementary material for: Association of Radioactive Iodine, Antithyroid Drug, and Surgical Treatments With Solid Cancer Mortality in Patients With Hyperthyroidism
Source: JAMA Netw Open. 2020 Jul 23;3(7):e209660. doi: 10.1001/jamanetworkopen.2020.9660 (PMC7378755; doi:10.1001/jamanetworkopen.2020.9660)
Supplement: Supplement. — eTable 1. Standardized Mortality Ratios (SMRs) and 95% Confidence Intervals (CIs) for Solid Cancer Mortality by Treatment Combination, Based on Ratio of Observed Numbers of Deaths Among Patients With Hyperthyroidism Versus Expected Numbers of Deaths in the General Population, by Time Between Study Entry and Death eTable 2. Standardized Mortality Ratios (SMRs) by Treatment Combination, Based on Ratio of Observed Numbers of Deaths Among Patients With Hyperthyroidism Versus Expected Numbers of Deaths in the General Population, Excluding Patients With Prior Cancers [file jamanetwopen-3-e209660-s001.pdf]

Supplementary Online Content

Kitahara CM, Preston DL, Sosa JA, Berrington de Gonzalez A. Association of radioactive iodine, antithyroid drug, and surgical treatments with solid cancer mortality in patients with hyperthyroidism. *JAMA Netw Open*. 2020;3(7):e209660. doi:10.1001/jamanetworkopen.2020.9660

eTable 1. Standardized Mortality Ratios (SMRs) and 95% Confidence Intervals (CIs) for Solid Cancer Mortality by Treatment Combination, Based on Ratio of Observed Numbers of Deaths Among Patients With Hyperthyroidism Versus Expected Numbers of Deaths in the General Population, by Time Between Study Entry and Death

eTable 2. Standardized Mortality Ratios (SMRs) by Treatment Combination, Based on Ratio of Observed Numbers of Deaths Among Patients With Hyperthyroidism Versus Expected Numbers of Deaths in the General Population, Excluding Patients With Prior Cancers

This supplementary material has been provided by the authors to give readers additional information about their work.

© 2020 Kitahara CM et al. *JAMA Network Open.*

eTable 1. Standardized mortality ratios (SMRs) and 95% confidence intervals (CIs) for solid cancer mortality by treatment combination, based on ratio of observed numbers of deaths among patients with hyperthyroidism versus expected numbers of deaths in the general population, by time between study entry and death

	Surgery only	Drugs only	Surgery + drugs	RAI only	Surgery + RAI	Drugs + RAI	Drugs + surgery + RAI
**Full cohort**							
<5 years							
Observed deaths	13	55	65	142	8	174	39
SMR (95% CI)	1.23 (0.71-2.11)	5.30 (4.07-6.91)	0.87 (0.68-1.11)	1.54 (1.31-1.81)	0.91 (0.45-1.81)	1.44 (1.24-1.67)	1.20 (0.88-1.64)
5-9 years							
Observed deaths	16	15	92	103	9	140	24
SMR (95% CI)	1.34 (0.82-2.19)	1.56 (0.94-2.59)	0.99 (0.81-1.21)	1.12 (0.92-1.36)	1.02 (0.53-1.96)	1.23 (1.05-1.46)	0.91 (0.61-1.36)
10-19 years							
Observed deaths	23	17	205	202	15	252	51
SMR (95% CI)	0.91 (0.61-1.38)	0.83 (0.51-1.33)	0.90 (0.78-1.03)	1.00 (0.87-1.14)	0.77 (0.46-1.27)	1.02 (0.90-0.15)	0.84 (0.64-1.10)
≥20 years							
Observed deaths	47	62	920	499	36	589	168
SMR (95% CI)	0.73 (0.55-0.97)	0.84 (0.66-1.08)	0.91 (0.86-0.98)	0.92 (0.84-1.01)	0.62 (0.45-0.86)	0.95 (0.87-1.03)	0.90 (0.78-1.05)
**Excluding patients with baseline history of cancer**							
<5 years							
Observed deaths	6	11	39	59	2	80	19
SMR (95% CI)	0.59 (0.26-1.31)	1.14 (0.63-2.05)	0.54 (0.39-0.74)	0.69 (0.56-0.86)	0.23 (0.06-0.94)	0.67 (0.52-0.86)	0.61 (0.39-0.96)
5-9 years							
Observed deaths	14	12	81	92	9	117	22
SMR (95% CI)	1.21 (0.71-2.04)	1.29 (0.73-2.28)	0.89 (0.72-1.11)	1.07 (0.89-1.28)	1.04 (0.54-2.00)	1.04 (0.84-1.27)	0.87 (0.57-1.32)
10-19 years							
Observed deaths	23	17	196	185	15	235	47
SMR (95% CI)	0.93 (0.62-1.40)	0.85 (0.53-1.36)	0.88 (0.76-1.01)	0.98 (0.86-1.11)	0.78 (0.47-1.29)	0.93 (0.81-1.08)	0.79 (0.60-1.05)
≥20 years							
Observed deaths	47	61	906	482	36	577	167
SMR (95% CI)	0.74 (0.55-0.98)	0.83 (0.65-1.07)	0.91 (0.85-0.97)	0.95 (0.88-1.03)	0.63 (0.45-0.87)	0.90 (0.82-0.99)	0.90 (0.77-1.05)

CI= confidence interval; RAI= radioactive iodine; SMR= standardized mortality ratio

[a] Using attained age, sex, calendar period (five-year), country (United States and United Kingdom) specific strata

© 2020 Kitahara CM et al. *JAMA Network Open.*

eTable 2. Standardized mortality ratios (SMRs) by treatment combination, based on ratio of observed numbers of deaths among patients with hyperthyroidism versus expected numbers of deaths in the general population, excluding patients with prior cancers

	**Surgery only**	**Drugs only**	**Surgery + drugs**	**RAI only**	**Surgery + RAI**	**Drugs + RAI**	**Drugs + surgery + RAI**
**Oral cavity**							
Observed deaths	1	4	20	12	3	18	1
SMR (95% CI)	0.55 (0.08-3.94)	2.38 (0.89-6.34)	0.93 (0.60-1.44)	0.78 (0.45-1.38)	2.00 (0.65-6.21)	0.99 (0.63-1.58)	0.21 (0.03-1.51)
**Esophagus**	2	3	16	20	1	12	5
Observed deaths	1.19 (0.30-4.76)	1.71 (0.55-5.32)	0.74 (0.46-1.21)	1.31 (0.85-2.03)	0.69 (0.10-4.90)	0.67 (0.38-1.18)	1.09 (0.45-2.62)
SMR (95% CI)							
**Stomach**	6	6	57	45	1	53	9
Observed deaths	1.01 (0.45-2.24)	1.10 (0.50-2.46)	0.99 (0.76-1.28)	1.14 (0.85-1.53)	0.25 (0.03-1.76)	1.11 (0.85-1.45)	0.70 (0.36-1.35)
SMR (95% CI)							
**Colon**	7	12	151	104	8	133	42
Observed deaths	0.45 (0.21-0.94)	0.86 (0.49-1.52)	0.88 (0.75-1.03)	0.90 (0.74-1.09)	0.64 (0.32-1.28)	0.97 (0.82-1.15)	1.08 (0.80-1.46)
SMR (95% CI)							
**Rectum**	3	0	28	28	0	20	7
Observed deaths	0.82 (0.27-2.55)	--	0.77 (0.53-1.11)	1.12 (0.77-1.62)	--	0.97 (0.82-1.15)	0.85 (0.40-1.78)
SMR (95% CI)							
**Liver**	3	3	45	43	3	38	11
Observed deaths	0.96 (0.31-2.99)	1.12 (0.36-3.49)	1.36 (1.02-1.83)	2.00 (1.49-2.70)	1.36 (0.44-4.21)	1.51 (1.10-2.07)	1.55 (0.86-2.80)
SMR (95% CI)							
**Pancreas**	7	4	89	55	5	72	16
Observed deaths	1.00 (0.48-2.10)	0.58 (0.22-1.55)	1.04 (0.84-1.27)	0.97 (0.74-1.26)	0.85 (0.35-2.03)	1.08 (0.86-1.36)	0.85 (0.52-1.39)
SMR (95% CI)							
**Lung/bronchus**							
Observed deaths	16	18	289	161	13	223	59
SMR (95% CI)	0.85 (0.52-1.38)	0.79 (0.50-1.26)	0.99 (0.88-1.11)	0.83 (0.71-0.97)	0.72 (0.42-1.23)	0.98 (0.86-1.12)	0.98 (0.76-1.27)
**Bladder**							
Observed deaths	1	3	25	23	1	33	2
SMR (95% CI)	0.32 (0.04-2.26)	1.12 (0.36-3.49)	0.74 (0.50-1.10)	0.96 (0.63-1.44)	0.41 (0.06-2.88)	1.16 (0.82-1.63)	0.28 (0.07-1.10)
**Kidney**							
Observed deaths	1	3	26	15	3	27	6
SMR (95% CI)	0.48 (0.07-3.39)	1.47 (0.47-4.56)	0.98 (0.67-1.44)	0.83 (0.50-1.38)	1.66 (0.53-5.14)	1.29 (0.88-1.88)	1.04 (0.47-2.32)

© 2020 Kitahara CM et al. *JAMA Network Open.*

**Brain/central nervous system**							
Observed deaths	2	4	27	18	1	21	2
SMR (95% CI)	1.10 (0.28-4.41)	1.86 (0.70-4.96)	0.99 (0.68-1.44)	1.04 (0.65-1.64)	0.57 (0.08-4.01)	1.03 (0.67-1.58)	0.33 (0.08-1.33)
**Thyroid**							
Observed deaths	0	0	4	9	1	11	3
SMR (95% CI)	--	--	0.78 (0.29-2.09)	2.59 (1.35-4.98)	2.73 (0.38-19.4)	2.74 (1.52-4.95)	2.60 (0.84-8.05)
**Uterus**							
Observed deaths	4	2	33	24	2	38	8
SMR (95% CI)	0.74 (0.28-1.96)	0.42 (0.11-1.68)	0.56 (0.40-0.79)	0.68 (0.45-1.01)	0.49 (0.12-1.97)	0.91 (0.67-1.26)	0.58 (0.29-1.16)
**Ovary**							
Observed deaths	6	3	54	45	0	56	10
SMR (95% CI)	1.16 (0.52-2.59)	0.54 (0.18-1.69)	0.79 (0.61-1.04)	1.06 (0.79-1.42)	--	1.15 (0.89-1.50)	0.64 (0.34-1.19)
**Prostate**							
Observed deaths	1	3	32	22	2	30	2
SMR (95% CI)	0.26 (0.04-1.88)	1.04 (0.34-3.22)	0.77 (0.54-1.09)	0.63 (0.41-0.96)	0.59 (0.15-2.36)	0.71 (0.50-1.01)	0.25 (0.06-1.00)

CI= confidence interval; RAI= radioactive iodine; SMR= standardized mortality ratio

[a]Using attained age, sex, calendar period (five-year), country (United States and United Kingdom) specific strata

© 2020 Kitahara CM et al. *JAMA Network Open.*
